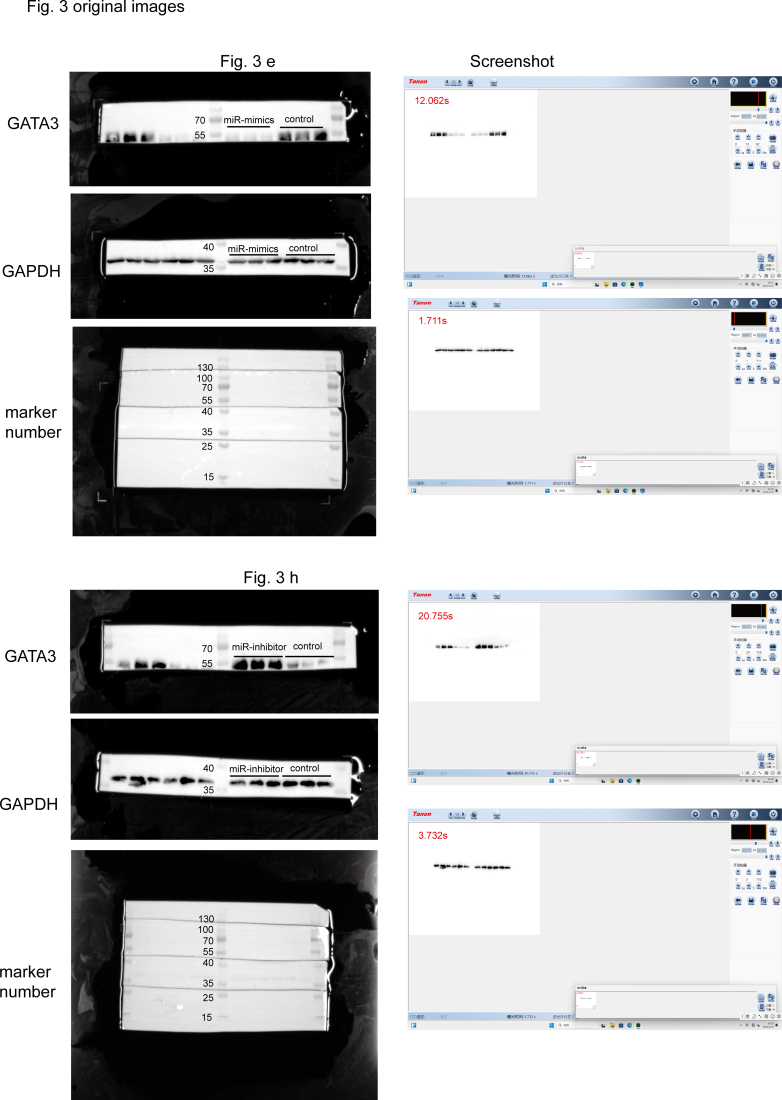# LncRNA PELATON regulates Th2 cell differentiation by miR-10b-5p/GATA3 axis in allergic rhinitis^☆^

**DOI:** 10.1016/j.waojou.2025.101108

**Published:** 2025-08-21

**Authors:** Zhen Liu, Yumei Li, Xiangkun Zhao, Qi Sun, Yu Zhang, Yaqi Wang, Han Fang, Yujuan Yang, Yakui Mou, Xicheng Song

**Affiliations:** a Department of Otorhinolaryngology, Head and Neck Surgery, Yantai Yuhuangding Hospital, Qingdao University, Yantai 264000, China; b Shandong Provincial Clinical Research Center for Otorhinolaryngologic Diseases, Yantai 264000, China; c The Second School of Clinical Medicine of Binzhou Medical University, Yantai 264000, China

**Keywords:** Allergic rhinitis, RNA, Long noncoding, GATA3 transcription factor, MicroRNAs, Th2 cells

## Abstract

**Background:**

Long non-coding RNAs (lncRNAs) have been increasingly recognized as critical regulators in the pathogenesis of immunoinflammatory diseases. Nevertheless, their mechanistic contributions to Th2-driven allergic rhinitis (AR) remain unclear. This study investigated the immunomodulatory function of lncRNA PELATON in AR pathogenesis by exploring its regulatory effects on Th2 cell differentiation.

**Methods:**

A total of 32 participants were enrolled, comprising 16 patients diagnosed with AR and 16 control subjects. The allergy levels of the participants were assessed using the skin prick test (SPT), and the levels of PELATON expression in PBMCs were detected by qPCR. Spearman's correlation analysis was used to calculate the correlation between the 2 variables. Changes in GATA3, IL-4, IL-5, and IL-13 expression were examined, as well as changes in Th2 cell differentiation resulting from the overexpression of PELATON and miR-10b-5p. Modulation of miR-10b-5p and GATA3 expression by PELATON was studied via transcriptome sequencing, dual-luciferase reporter assays, and *in vitro* experiments.

**Results:**

LncRNA PELATON expression was elevated in patients with AR and showed a significant correlation with allergy levels. PELATON was enriched in CD4^+^T cells. Overexpression of PELATON in PBMCs led to an increase in the proportion of Th2 cells and the levels of GATA3, IL-4, IL-5, and IL-13 expression, while the overexpression of miR-10b-5p reduced the expression levels of GATA3, IL-4, IL-5, and IL-13. PELATON bound directly to miR-10b-5p and inhibited the negative regulatory effect of miR-10b-5p on GATA3, thereby forming a ceRNA network that promoted Th2 cell differentiation.

**Conclusion:**

LncRNA PELATON competitively binds to miR-10b-5p and promotes GATA3 expression, resulting in Th2 cell differentiation and activation in AR. Formation of the lncRNA PELATON/miR-10b-5p/GATA3 ceRNA network is a regulatory mechanism that offers new possibilities for finding therapeutic targets for AR.

## Introduction

Allergic rhinitis (AR) is an IgE-mediated nasal mucosal inflammation caused by allergens and presents with symptoms of rhinorrhea, sneezing, nasal congestion, and itching. AR affects 10–30% of adults globally, and approximately 40% of AR patients subsequently develop asthma, which imposes a substantial economic burden on society.^1^ The current treatments for AR include avoiding contact with allergens, surgical intervention, specific immunotherapy, and the use of pharmacotherapy, such as H1-receptor blockers, nasal glucocorticoids, and leukotriene receptor antagonists. However, relapse after medication withdrawal and local or systemic adverse reactions have been reported.^2^ Therefore, it is crucial to clarify the molecular mechanism of AR and discover new therapeutic targets.

Accumulating evidence indicates that dysregulated differentiation of CD4^+^T lymphocytes into Th1 and Th2 subsets is a key immunological factor that contributes to AR pathogenesis.^3^ Allergens are processed by cells and subsequently presented to CD4^+^T cells, leading to increased expression of GATA3. This transcription factor drives Th2 polarization characterized by elevated levels of IL-4, IL-5, and IL-13 that contribute to AR pathogenesis.^4^^,^^5^

Long noncoding RNAs (lncRNAs) are RNA molecules exceeding 200 nucleotides in length. They can be categorized based on their relative positions to known neighboring "protein-encoding" exons. LncRNAs transcribed from regions between protein-encoding genes are known as long intergenic lncRNAs, whereas those transcribed from intronic regions are called intronic lncRNAs.^6^ LncRNAs function as transcriptional regulators via their direct interactions with chromatin-modifying proteins and transcription factors.^7^ The functions of lncRNAs are highly dependent on subcellular localization.^8^ Numerous studies have demonstrated that lncRNAs are critically involved in the pathogenesis of AR through their regulatory effects on immune responses, and particularly by modulating the differentiation of B and T lymphocytes.^9^ Previous studies have reported the dysregulated expression of 2259 lncRNAs in the nasal mucosa of patients with AR, and a lncRNA-mRNA co-expression analysis showed that lncRNAs in the nasal mucosa and peripheral blood participate in AR pathogenesis via different pathways.^10^ PELATON, which is also known as LINC01272 and ENST00000445003.1, is a long non-coding RNA located at the 50,247,487–50,287,035 site of chromosome 20.^11^ Previous studies found that the LINC01272 (PELATON)/miR-876/ITGB2 axis promotes colorectal cancer metastasis^12^ and induces cell apoptosis.^13^^,^^14^ In addition, LINC01272 is a potential new biomarker for certain immune diseases, including inflammatory bowel disease and Crohn's disease.^15^^,^^16^ Our previous study found that PELATON was highly expressed in the peripheral blood of patients with AR.^17^ Recent studies have revealed that lncRNAs function as molecular sponges for microRNAs (miRNAs) in the cytoplasm.^18^^,^^19^ This mechanism is mediated by the binding of miRNAs to complementary sequences within the 3' untranslated regions (UTRs) of target mRNAs, thereby establishing a competitive endogenous RNA (ceRNA) network.^20^

Although several studies have demonstrated the differential expression of lncRNAs and miRNAs in patients with AR, the precise regulatory mechanisms of lncRNAs in AR pathogenesis remain poorly understood. Therefore, a thorough investigation into the function of lncRNA in AR could assist in identifying biomarkers that aid in the diagnosis, treatment, and prediction of AR. This study examined the pivotal role played by PELATON in Th2 cell differentiation within AR and provides critical insights into the underlying mechanisms that drive the pathogenesis of allergic airway diseases.

## Materials and methods

### Patient inclusion and exclusion criteria

This study enrolled a total of 32 participants, comprising 16 patients diagnosed with AR and 16 control subjects. All patients had a confirmed diagnosis of AR, as defined by the Chinese Guidelines for the Diagnosis and Treatment of Allergic Rhinitis.^21^ Patients with AR had to have clinical symptoms associated with an allergen and show a positive skin prick test (SPT) for that allergen. All patients with AR exhibited positive results for specific IgE testing and had abstained from any systemic medications, including corticosteroids, biologic therapy, or allergen-specific immunotherapy, for at least 3 months prior to enrollment. Individuals with concurrent nasal pathologies (eg, tumors, inflammatory conditions), systemic diseases, or other allergic disorders such as asthma, atopic dermatitis, or atopic colitis were excluded from enrollment. Control subjects were required to have negative specific IgE test results. The study received ethical approval from the Institutional Ethics Committee, and all subjects provided their written informed consent prior to enrollment. Detailed clinical characteristics of both the AR and control subjects are shown in Supplementary Table 1.

### Allergy detection

The allergic sensitization of participants in this study was evaluated using the SPT, which is a highly sensitive and standardized method used for detecting IgE-mediated type I hypersensitivity and diagnosing AR.^22^^,^^23^ The SPT reaction intensity was quantified using the skin index (SI), which is calculated as the ratio of the allergen-induced wheal diameter to a corresponding histamine-induced wheal diameter. Based on the SI values, the reactions were classified into 4 grades: + (0.3 ≤ SI < 0.5), ++ (0.5 ≤ SI < 1.0), +++ (1.0 ≤ SI < 2.0), and ++++ (SI ≥ 2.0).^24^

### Total Nasal Symptom Score

Total Nasal Symptom Score (TNSS) is defined as the sum of individual symptom scores for rhinorrhea, nasal congestion, nasal pruritus, and sneezing, with each symptom rated on a 4-point scale ranging from 0 (none) to 3 (severe).

### PBMC and CD4^+^T cell isolation and culture

Peripheral blood mononuclear cells (PBMCs) were isolated from freshly collected anticoagulated peripheral blood using a Ficoll density gradient centrifugation kit (Solarbio, China). Following isolation, the PBMCs were washed twice with phosphate-buffered saline (PBS). Subsequently, CD4^+^T cells were purified from the PBMCs using a human CD4^+^T Cell Isolation Kit (Miltenyi Biotec, Germany), according to the manufacturer's instructions. Prior to culture, the cells were pre-treated with 5 μg/mL anti-CD3 and anti-CD28 antibodies in 6-well plates to stimulate T cell activation. Both the PBMCs and CD4^+^T cells were then cultured in RPMI 1640 medium supplemented with 10% fetal bovine serum (FBS) at 37°C in a humidified 5% CO2 incubator.

### Separation of the cell nucleus and cytoplasm and PELATON expression analysis

Subcellular localization of PELATON was analyzed by performing nucleocytoplasmic fractionation assays. A PARIS™ Kit Protein and RNA Isolation System (Thermo Fisher Scientific, Waltham, MA, USA) was used to perform nuclear and cytoplasmic fractionation of Jurkat cells, which is an immortalized human T-lymphocyte cell line extensively utilized in biomedical research for investigating T-cell signaling pathways and the expression profiles of chemokine receptors.^25^ This was followed by RNA extraction from the nucleus and cytoplasm. The extracted RNA was subsequently reverse-transcribed into cDNA using an Evo M-MLV Mix Kit with gDNA Clean for qPCR Kit (Accurate Biotech, China). The cDNA amplification was performed using a 2 × EasyTaq PCR SuperMix (+Dye) (TransGen Biotech, Beijing) kit. PELATON expression in the nucleus and cytoplasm was detected using 2% agarose gel electrophoresis (100 V, 40 min).

### Construction and transfection of PELATON overexpression lentivirus

PBMCs and Jurkat cells were cultured for 24 h and then infected with an appropriate volume of control or PELATON overexpression lentivirus (constructed by Hanheng Biological Technology, MOI = 5), followed by the addition of 6 μg/mL polybrene. Purinomycin (1 μg/mL) was added to Jurkat cells at 48 h after infection to screen for stably transfected cell lines.

### Transcriptional small RNA sequencing and analysis

To identify PELATON-regulated downstream genes, we performed transcriptome sequencing using PELATON-overexpressing Jurkat cells, with sequencing services provided by Applied Protein Technology (Shanghai, China). Total RNA was extracted from the samples using Trizol Reagent (Invitrogen Life Technologies, Carlsbad, CA, USA). Small RNA libraries were constructed using the NEBNext Multiplex Small RNA Library Prep Set for Illumina. The quantified libraries were combined and sequenced on Illumina platforms based on the optimal library concentration and necessary data. The detailed steps are mentioned in a previous publication by Arindrarto et al, 2021.^26^

### Differential expression of miRNAs and target gene prediction

A differential expression analysis was conducted between the control group and the PELATON overexpression group by using the DESeq R package (version 1.8.3). The *P*-values were adjusted using the Benjamini-Hochberg method. The Benjamini-Hochberg method was employed to adjust the *P*-values, with a default significance threshold set at a corrected *P*-value of 0.05 for identifying differentially expressed genes. Additionally, the target genes of miRNAs were predicted using the miRanda (Enright et al., 2003).

The predicted target genes of the differentially expressed miRNAs were identified through a KEGG analysis (https://www.kegg.jp/). The target genes related to Th2 differentiation were screened based on the KEGG results, followed by the screening of Th2 differentiation-related miRNAs from differentially expressed miRNAs.

### Transfection of PELATON siRNA, miR-10b-5p inhibitors, and mimics

Specific siRNAs for PELATON, as well as inhibitors and mimics for miR-10b-5p, were designed and produced by GenePharma (Shanghai, China). The sequences used were as follows: PELATON siRNA: 5’-CAAGUGCCCAGUAAACACUTT-3’; miR-10b-5p inhibitor: 5′-CACAAAUUCGGUUCUACAGGGUA-3’; miR-10b-5p mimics: sense-5′- UACCCUGUAGAACCGAAUUUGUG-3′, antisense-5′-CAAAUUCGGUUCUACAGGGUAUU-3’. SiRNAs, inhibitors, and mimics were transfected into PBMCs at 48h by using Lipofectamine 3000 (Thermo Fisher Scientific, USA), according to the manufacturer's instructions.

### RNA extraction and qPCR

Changes in gene expression at the transcriptional level were quantified using qPCR. RNA was extracted from PBMC cells with SparkZol Reagent (SparkJade, China), according to the manufacturer's protocol. For lncRNA, reverse transcription into cDNA was carried out using a total-transcriptome cDNA Synthesis Kit (Applied Biological Materials, USA). Similarly, microRNAs were converted into cDNA by using a miRNA 1st Strand cDNA Synthesis Kit (Accurate Biotech, China), according to the provided guidelines. The 2 × SYBR Green QPCR Mix (With ROX) reagent (SparkJade, China) was used for performing qPCR, with GAPDH and U6 serving as the reference genes. Gene expression levels were quantified via the 2^−ΔΔCT^ method, and each experiment was conducted in triplicate. The mean values derived from the replicates were utilized for subsequent comparative analyses. The primers were synthesized by Sangon Biotech (China) and are shown in Supplementary Table 3.

### Protein extraction and western blotting

Changes in gene expression at the translational level were detected by western blotting. Total proteins were extracted from PBMC cells using RIPA lysis buffer (Solarbio, China). The protein samples were resolved via SDS-PAGE, and the protein bands were subsequently transferred onto PVDF membranes, which were blocked to prevent non-specific binding. Next, the membranes were incubated overnight at 4°C with primary antibodies that included rabbit anti-β-ACTIN, anti-GAPDH (1:1000, Affinity, Cincinnati, OH, USA), and anti-GATA3 (1:1000, Sino Biological, China); after which, the membranes were incubated with an HRP-conjugated goat anti-rabbit secondary antibody (1:1000, Affinity, USA) for 2 h at room temperature. Protein-antibody complexes were visualized using a hypersensitive chemiluminescent reagent (SparkJade, China). Protein expression levels were quantified using Image J software and normalized against those of GAPDH.

### Luciferase assay

Dual-luciferase reporter assays were performed to verify the binding sites between PELATON, miR-10b-5p, and GATA3. PELATON was predicted to bind to miR-10b-5p via the 26–30 5′-GGTTC-3′ site. In turn, miR-10b-5p was predicted to bind to the GATA3-3′ UTR region at site 236–242 5′-ACAGGG-3'. In order to block miR-10b-5p binding to PELATON or the GATA3-3′ UTR, we mutated the binding sites from 5′-GGTTC-3′ to 5′-AACCG-3′ in PELATON, and from 5′-ACAGGG-3′ to 5′-GCGAAA-3′ in the GATA3-3′ UTR. Next, we constructed psiCHECK2 vector plasmids (Sangon Biotech, China) that contained the wild-type (WT) and mutant (MUT) PELATON and GATA3 3′UTR region, and designated them as PELATON-WT, GATA3-3′-UTR-WT, PELATON-MUT, and GATA3-3′-UTR-MUT. Plasmids were separately co-transfected into 293T cells along with miR-10b-5p mimics or mimic control (GenePharma, China) by using Lipofectamine 3000 for 48 h. Luciferase activity was measured using a Dual Luciferase Reporter Assay Kit (Vazyme, China).

### ELISA

Inflammatory mediators in PBMCs were quantified by ELISA. The supernatant of the PBMC cell culture solutions was collected, and the concentrations of IL-4, IL-5, and IL-13 were detected using the ELISA kit (Jiyinmei Biotechnology, China), as per the manufacturer's instructions.

### Flow cytometric analysis

Changes in Th2 cell proportions were analyzed by flow cytometry. PBMCs were incubated on ice with anti-human CD4-Percp-Cy5.5 (Thermo Fisher Scientific, USA) for 1 h in the dark. For intracellular GATA3 staining, cells were permeabilized using a permeabilization/fixation buffer (Thermo Fisher Scientific, USA) and then labeled with anti-human-GATA3-AF488 (Thermo Fisher Scientific, USA) for 1 h. The stained samples were analyzed using a FACSCalibur flow cytometer (BD Biosciences, San Jose, CA, USA), and data were processed with FlowJo_v10.8.1 (FlowJo software, USA).

Gating strategy for sorting CD4^+^GATA3^+^ Th2 cells by flow cytometry: Mononuclear cells (10,000 cells) were gated from all cells according to SSC-A/FSC-A as subset-1. CD4^+^T cells stained with anti-human-CD4-Percp-Cy5.5 antibody were gated from subset-1. GATA3^+^ cells stained with anti-human-GATA3-AF488 antibody were gated from CD4^+^T cells.

### Correlation analysis

The correlation between PELATON expression levels in PBMCs and allergy levels, as well as TNSS, and the correlation between PELATON and miR-10b-5p expression levels were assessed using Spearman's correlation analysis.

### Statistical analysis

All data were analyzed using GraphPad Prism 9.0 software (GraphPad Software, USA), and results are expressed as a mean value ± standard deviation. Gene expression (qRT-PCR) was quantified using the 2^−ΔΔCT^ method and normalized to GAPDH and U6. All experiments were conducted with at least 3 replicates. Differences between groups were evaluated using the unpaired *t*-test (for 2 groups) or one-way ANOVA (for ≥ 3 groups) with Tukey's post hoc test, depending on normality,^27^ and A *P*-value <0.05 was considered to be statistically significant.

## Results

### PELATON was highly expressed in AR

PELATON expression was significantly increased in the peripheral blood of patients with AR when compared to healthy control subjects (Fig. 1a). PELATON expression was significantly correlated with allergic levels and TNSS, as shown by the Spearman's rank correlation coefficient (Fig. 1b and c, Supplementary Table 2). CD4^+^T cells and non-CD4^+^T cells were isolated from PBMCs. PELATON expression was markedly elevated in CD4^+^T cells when compared to non-CD4^+^T cells (Fig. 1d). We separated the Jurkat cell nucleus from the cytoplasm before studying the PELATON expression levels and detected PELATON expression in both the nucleus and cytoplasm (Fig. 1e). Our results suggest that PELATON could serve as a key immunoregulatory factor within PBMCs, and particularly in the CD4^+^T lymphocytes of patients with AR.

**Fig. 1:**
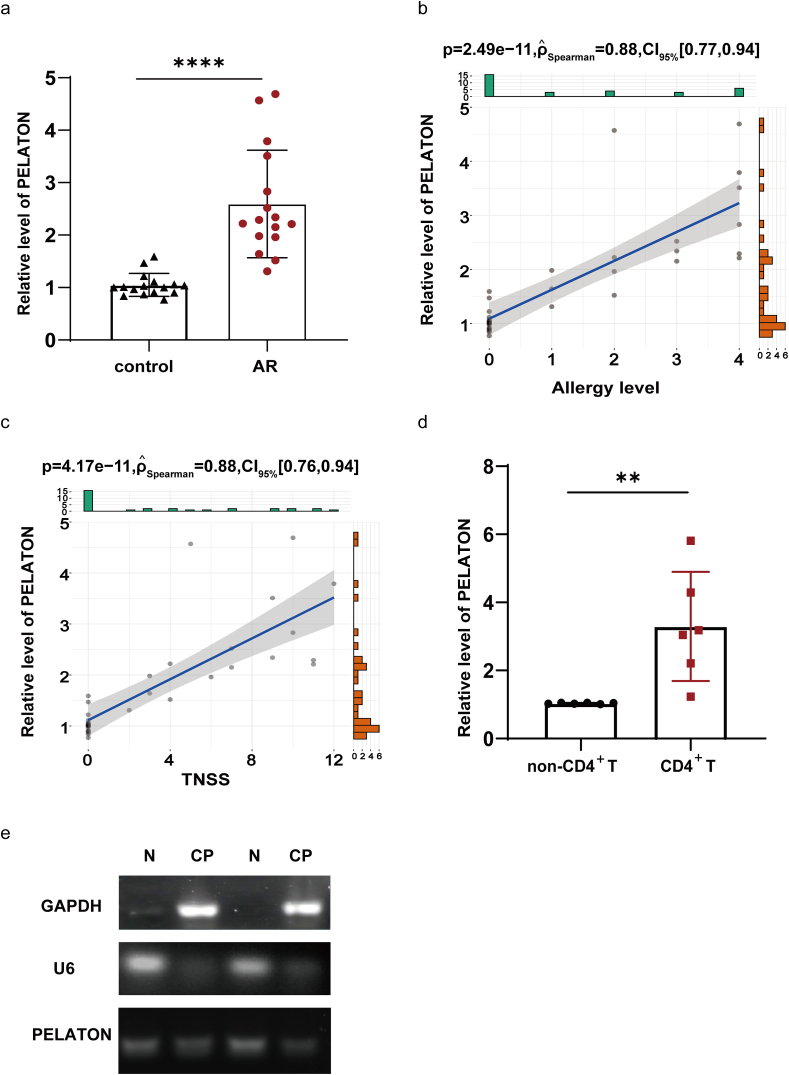
Expression of PELATON in AR. (a) Expression of PELATON in AR (n = 16) and control subjects (n = 16) was detected by qPCR. (b, c) Correlation analysis of the relative expression of PELATON with the corresponding allergy level and Total Nasal Symptom Score (TNSS) in all subjects by using the Spearman's rank correlation coefficient. The vertical coordinate is the relative expression level of PELATON and the horizontal coordinate is the corresponding allergy level or TNSS. N = 32. (d) Expression of PELATON in non-CD4^+^T cells (n = 6) vs. CD4^+^T (n = 6) cells was detected by qPCR. (e) Detection of PELATON expression in the intracellular nucleus and cytoplasm after nucleoplasmic separation in Jurkat cells. N, nucleus, CP, cytoplasm. ∗∗, *P* < 0.01

### PELATON promoted the differentiation and activation of Th2 cells

To explore the effect of PELATON on Th2 cell differentiation, we forced the overexpression and knockdown of PELATON in PBMCs, and then detected the expression levels of the Th2 cell-specific transcription factor GATA3 and the inflammatory factors IL-4, IL-5, and IL-13 in the PBMCs. Overexpression of PELATON in PBMCs (Fig. 2a) was significantly enhanced the expression of GATA3 (Fig. 2b, 2c, 2d and Supplementary Fig. 2), upregulated the production of Th2-associated cytokines, including IL-4, IL-5, and IL-13 (Fig. 2e, 2f, 2g). Knockdown of PELATON in PBMCs (Fig. 2h) significantly reduced the expression levels of GATA3 (Fig. 2i, 2j, 2k, and Supplementary Fig. 2), IL-4, IL-5, and IL-13 (Fig. 2l, 2m, 2n). A significant increase in the proportion of Th2 cells in the CD4^+^T cell population was detected by flow cytometry after the overexpression of PELATON (Fig. 2o, 2p, 2q, and Supplementary Fig. 1). These results suggest that PELATON promotes the differentiation of CD4^+^T cells toward the Th2 lineage.

**Fig. 2:**
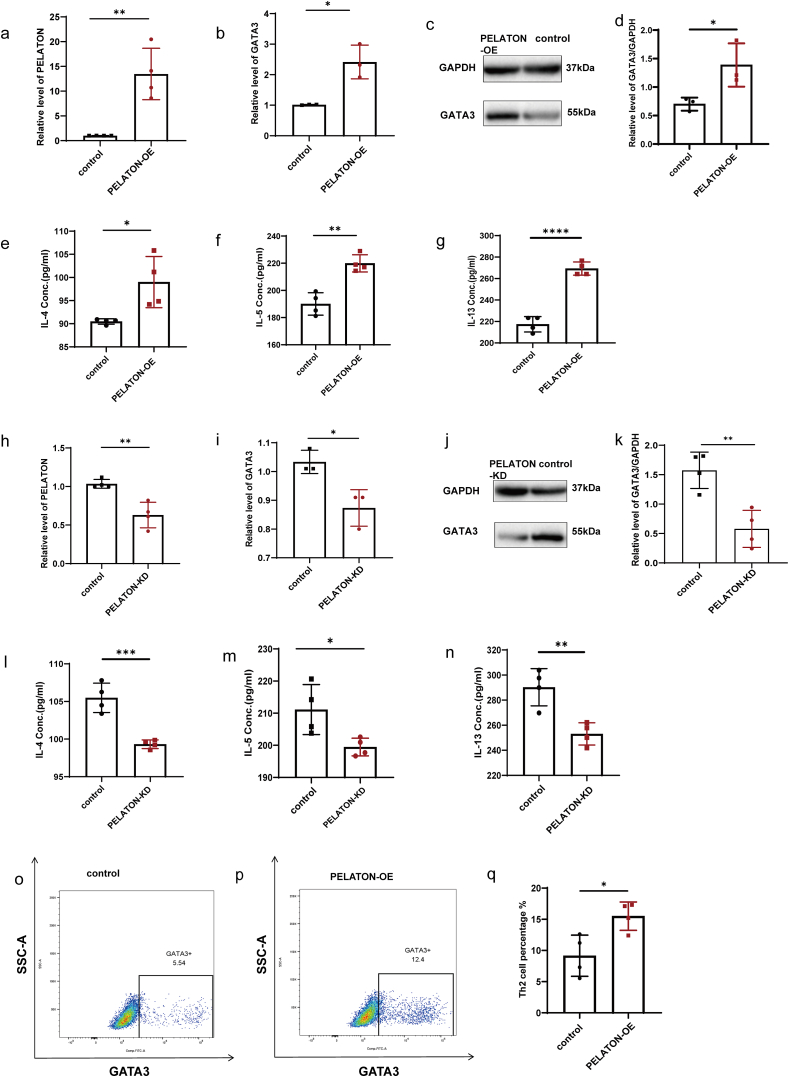
Effect of PELATON on Th2 cell differentiation and activation. (a) The efficiency of PELATON overexpression was detected by qPCR. (b) The expression of GATA3 mRNA in the control and PELATON overexpression groups was detected by qPCR. (c, d) The expression of GATA3 protein in the control and PELATON overexpression groups was detected by western blotting; the relative expression of GATA3/ACTIN was analyzed using ImageJ software (n = 4). (e, f, g) The expression of IL-4, IL-5, and IL-13 in the control and PELATON overexpression groups were detected by ELISA (n = 4). (h) The efficiency of PELATON knockdown was detected by qPCR. (i) The expression of GATA3 mRNA in the control and PELATON knockdown groups was detected by qPCR. (j, k) Expression of GATA3 protein in the control and PELATON knockdown groups was detected by western blotting; the relative expression of GATA3/ACTIN was analyzed using ImageJ software. N = 4. (l, m, n) The expression of IL-4, IL-5, and IL-13 in the control and PELATON knockdown groups were detected by ELISA (n = 4). Flow cytometry was used to detect (o) the proportion of GATA3+Th2 cells in the CD4+T cell population of the control group, and (p) the proportion of GATA3+Th2 cells in the CD4+T cell population of the PELATON overexpression group. (q) A comparative analysis of the Th2 cell proportion in the control group and PELATON overexpression group was performed using FlowJo software (n = 4). ∗, *P* < 0.05, ∗∗, *P* < 0.01, ∗∗∗, *P* < 0.001

### MiR-10b-5p decreased the differentiation and activation of Th2 cells

**Fig. 3:**
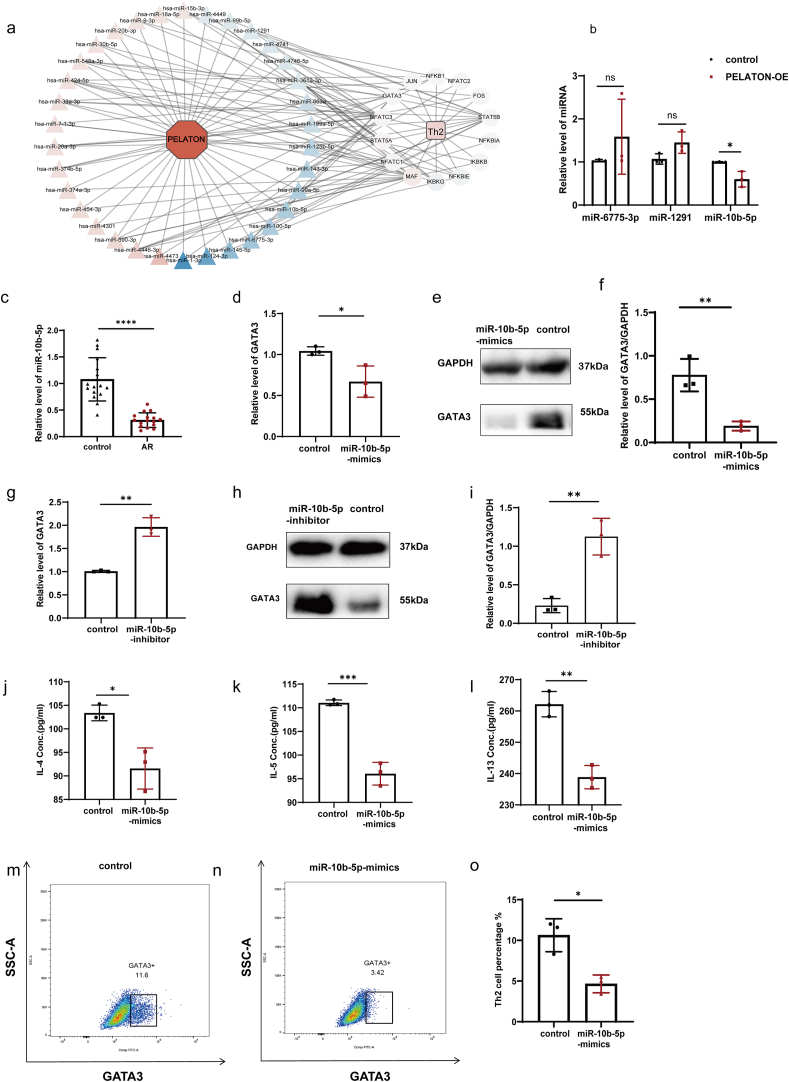
Effect of miR-10b-5p on Th2 cell differentiation and activation. (a) The regulatory relationship of 34 miRNAs and their 14 target mRNAs related to Th2 cell differentiation when PELATON was overexpressed in Jurkat cells. A qPCR-based analysis showed that: (b) MiR-10b-5p expression was significantly downregulated, and the expression of 2 other miRNAs was not significantly changed by PELATON overexpression, and (c) the expression of miR-10b-5p in AR (n = 16) and control subjects (n = 16). (d) Expression of GATA3 mRNA in the control and miR-10b-5p mimics groups, as detected by qPCR. (e) Expression of GATA3 protein in control versus miR-10b-5p mimic groups, as detected by western blotting. (f) The relative expression of GATA3/ACTIN was analyzed using ImageJ software (n = 4). (g) Expression of GATA3 mRNA in the control and miR-10b-5p inhibitor groups was detected by qPCR. (h) Expression of GATA3 protein in the control and miR-10b-5p inhibitor groups was detected by western blotting. (i) The relative expression of GATA3/ACTIN was analyzed using ImageJ software (n = 3). (j, k, l) Changes in the levels of IL-4, IL-5, and IL-13 in the cell supernatants of the control group versus the miR-10b-5p mimic groups, as detected by ELISA. (m, n) Proportion of GATA3^+^Th2 cells in the CD4^+^T cell population of the control and miR-10b-5p mimic groups, as detected by flow cytometry. (o) Comparison of Th2 cell proportions between the control and miR-10b-5p mimic groups as detected using FlowJo software (n = 3). ∗, *P* < 0.05, ∗∗, *P* < 0.01, ∗∗∗, *P* < 0.001

Previous studies reported that lncRNAs expressed in the cytoplasm often serve as molecular sponges for miRNAs that regulate gene expression.^28^^,^^29^ Our study revealed that PELATON was significantly expressed in the cytoplasm. In order to explore whether PELATON regulates Th2 cell differentiation by binding to miRNAs, we used transcriptome sequencing to detect changes that occurred in miRNA expression during PELATON overexpression, and found significant changes in the expression of all 51 miRNAs (Supplementary Table 4, P < 0.05). The 51 miRNAs collectively acted on a total of 88,171 target genes, as predicted by miRanda (Supplementary Table 5). Subsequently, a KEGG enrichment analysis was conducted on the target genes associated with the 51 miRNAs (Supplementary Table 6). The results revealed that 14 target genes of 34 miRNAs were significantly enriched in pathways related to the regulation of Th2 differentiation (Fig. 3a). Our data showed that among the 6 miRNAs predicted to bind to GATA3, 3 miRNAs (miR-6775-3P, miR-1291, and miR-10b-5p) were downregulated by PELATON overexpression. This was opposite to the up-regulated expression trend of GATA3, suggesting that the miRNAs might be GATA3 regulators. The other 3 miRNAs were upregulated by PELATON overexpression, which had a similar trend of expression with GATA3 and were opposite to the miRNA inhibition function on mRNAs. Hence, we only verified the expression of the 3 miRNAs, miR-6775-3P, miR-1291, and miR-10b-5p. The result showed that while miR-10b-5p expression was obviously downregulated, the levels of miR-6775-3p and miR-1291 expression did not significantly change in PBMCs that overexpressed PELATON (Fig. 3b).

We then verified the expression and function of miR-10b-5p in regulating Th2 in AR. In patients with AR, PBMCs exhibited a significantly reduced expression of miR-10b-5p when compared to PBMCs from control subjects (Fig. 3c). Overexpression of miR-10b-5p suppressed GATA3 expression (Fig. 3d, 3e, 3f, and Supplementary Fig. 3), whereas knockdown of miR-10b-5p led to an upregulation of GATA3 at both the mRNA and protein levels (Fig. 3g, 3h, 3i and Supplementary Fig. 3). These results showed that miR-10b-5p inhibited GATA3 expression. In addition, miR-10b-5p mimics were found to decrease the secretion of IL-4, IL-5, and IL-13 (Fig. 3j, 3k, and 3l). A flow cytometry analysis showed a significant decrease in the proportion of Th2 cells within the CD4^+^T cell population following miR-10b-5p overexpression (Fig. 3m, 3n, 3o). These findings collectively demonstrated that miR-10b-5p negatively regulates the differentiation and activation of Th2 cells.

### PELATON directly bound to miR-10b-5p to regulate GATA3 expression

**Fig. 4:**
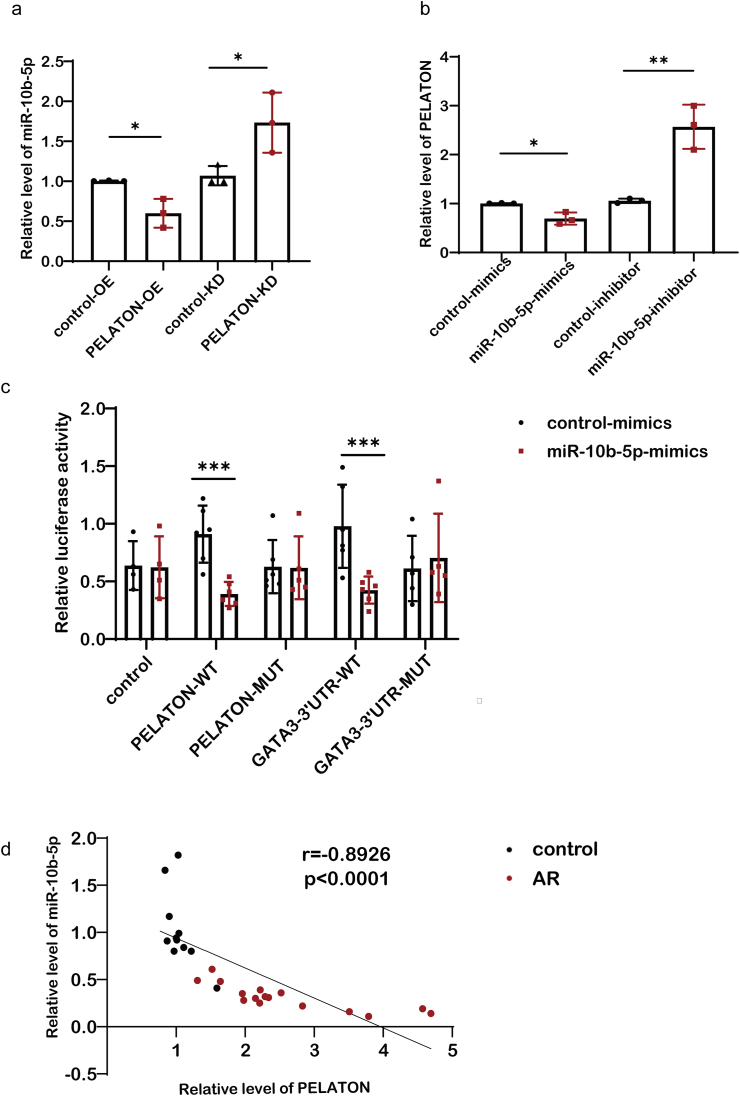
Binding of MiR-10b-5p to PELATON and GATA3-3′UTR. A qPCR-based analysis was performed of: (a) Expression of miR-10b-5p after PELATON overexpression or knockdown, and (b) Expression of PELATON after miR-10b-5p mimics or inhibitor treatment. (c) Relative luciferase activity of the PELATON WT/MUT and GATA3-3′UTR WT/MUT in the control and miR-10b-5p mimic groups (n = 5). ∗, *P* < 0.05, ∗∗, *P* < 0.01, ∗∗∗, *P* < 0.001. (d) Correlation analysis of the relative expression of PELATON with the corresponding miR-10b-5p levels in all subjects as determined by Spearman's rank correlation coefficient. n = 32, r = −0.8926, *P* < 0.0001

We next explored the association between PELATON and miR-10b-5p and found that miR-10b-5p expression decreased after PELATON was overexpressed and increased after PELATON knockdown (Fig. 4a). Similarly, miR-10b-5p overexpression decreased PELATON expression, and miR-10b-5p knockdown enhanced PELATON levels (Fig. 4b). The results suggest that PELATON might bind to miR-10b-5p. We hypothesized that PELATON might regulate GATA3 expression by directly binding to miR-10b-5p, thereby affecting Th2 cell differentiation and activation. Because miR-10b-5p might bind to PELATON at the 5′-GGTTC-3′ site and to GATA3-3′UTR at the 5′-ACAGGG-3′ site, we performed a dual luciferase reporter assay to verify our hypothesis. Subsequent results showed that miR-10b-5p mimics significantly reduced the relative luciferase activity of PELATON-WT, while no change was observed in the PELATON-MUT, thereby suggesting that miR-10b-5p can directly bind to PELATON (Fig. 4c). A similar trend was observed in the GATA3-3′UTR, which indicated that miR-10b-5p could also bind to the GATA3-3′UTR (Fig. 4c). Furthermore, the levels of PELATON and miR-10b-5p expression showed a significant negative correlation in both the AR and control subjects (r = −0.8926, *p* < 0.0001) (Fig. 4d). These results demonstrated that miR-10b-5p was capable of directly interacting with PELATON and the GATA3-3′UTR, thereby regulating their expression and function.

### MiR-10b-5p modulated the regulation of Th2 cell activation by PELATON

**Fig. 5:**
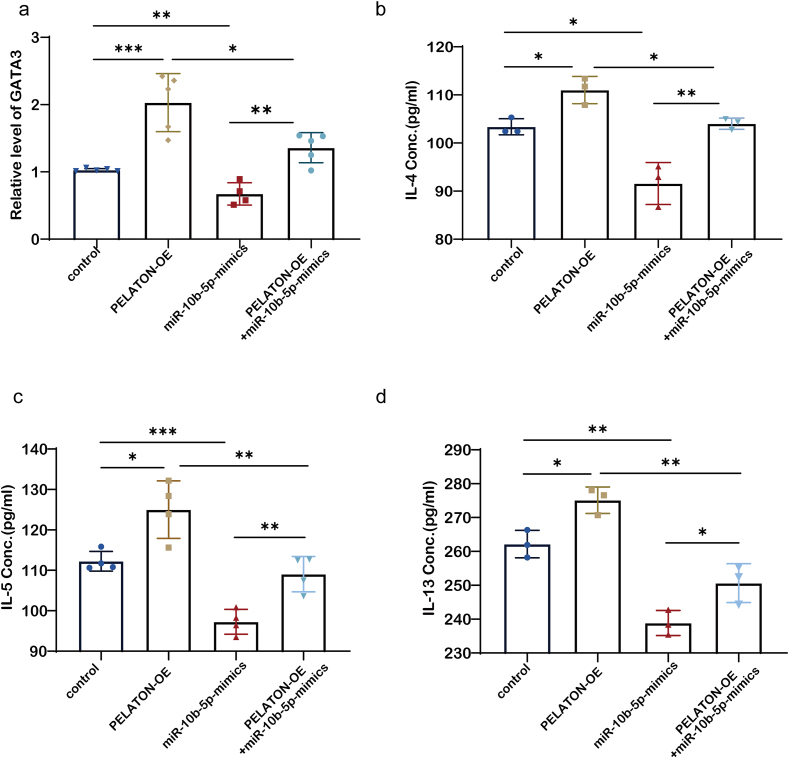
MiR-10b-5p attenuated the effect of PELATON on Th2 differentiation and activation. (a) Expression of GATA3 in the control group, PELATON overexpression group, miR-10b-5p mimics group, and PELATON and miR-10b-5p co-overexpression group, as detected by qPCR (n = 5). (b, c, d) Expression of IL-4, IL-5, and IL-13 in cell culture supernatants of the control group, PELATON overexpression group, miR-10b-5p mimics group, and PELATON and miR-10b-5p co-overexpression group, as detected by ELISA (n = 3). ∗, *P* < 0.05, ∗∗, *P* < 0.01, ∗∗∗, *P* < 0.001

To investigate the role of miR-10b-5p in PELATON-mediated regulation of Th2 cell differentiation, we examined the expression levels of the Th2-specific transcription factor GATA3 and the Th2-associated cytokines (IL-4, IL-5, and IL-13) in PBMCs following miR-10b-5p and PELATON overexpression. When both miR-10b-5p and PELATON were overexpressed, the expression levels of GATA3 and the 3 cytokines were lower than those when PELATON alone was overexpressed; however, the levels were higher than those when miR-10b-5p alone was overexpressed (Fig. 5a, 5b, 5c, and 5d). These results revealed that miR-10b-5p effectively inhibited the upregulation of GATA3, IL-4, IL-5, and IL-13 induced by PELATON overexpression. They further demonstrated that miR-10b-5p could directly bind to PELATON, thereby impairing the regulatory effect of PELATON on GATA3. Our results showed that PELATON acts as a competitive endogenous RNA (ceRNA) for miR-10b-5p, thereby modulating GATA3 expression. This regulatory mechanism subsequently influences the differentiation and activation of Th2 cells.

## Discussion

Th2 cell differentiation plays a crucial role in AR progression, with the ectopic accumulation of Th2 cells in AR leading to the release of Th2 cytokines, such as IL-4, IL-5, and IL-13.^30^ These cytokines trigger B cell proliferation and differentiation into plasma cells, resulting in the production of IgE and the initiation of type 2 allergic reaction.^31^ IL-4 plays a crucial role in driving the transformation of naïve CD4^+^T cells into Th2 cells.^32^ The inhibition of Th2 cell responses attenuates allergic inflammation in AR.^33^

Previous studies have demonstrated that lncRNAs play a critical role in CD4^+^ T cell differentiation and their dysregulated activation during allergic airway inflammatory diseases. LncRNA-PVT1 demonstrated significant positive associations with Th2 cell-mediated immune responses in patients with AR.^34^ Meanwhile, lncRNA-NEAT1 was strongly correlated with both clinical severity measures and systemic inflammation markers.^35^^,^^36^ Additionally, several lncRNAs were shown to be differentially expressed in CD4^+^T cells in a mouse model of allergic airway inflammation. Reversing the expression of those lncRNAs could effectively suppress the secretion of pro-inflammatory mediators and alleviate the symptoms of allergic inflammation. For instance, Ma et al. 2022 found that lncRNA FR215775 knockout in an AR mouse model alleviated allergic symptoms and reduced IL-4 and IL-5 expression.^37^ Our previous study also showed the involvement of lncRNA FAM239A in regulating Th cell responses.^38^ Our current study revealed that PELATON promotes the release of inflammatory factors resulting from an increase in Th2 cells in patients with AR. Moreover, this suggests that PELATON may be an AR regulator, which warrants further clinical investigation into its role as a biomarker of AR.

LncRNAs perform different functions based on their cellular localization. In the cytoplasm, lncRNAs modulate mRNAs by regulating miRNAs. This regulatory network influences diverse biological processes, such as cellular proliferation, differentiation, apoptosis, and activation, ultimately contributing to the development and progression of various diseases.^8^ However, it remains unknown whether lncRNAs can regulate Th2 cell responses by modulating miRNA function in AR.

Emerging evidence highlights the pivotal regulatory roles played by miRNAs in AR. For example, miR-135a, miR-124-3p, and miR-182-5p were reported to reduce the instances of nasal rubbing and sneezing in AR mouse models. They also reduced the levels of GATA3 and Th2 inflammatory factors, which are key to restoring the Th1/Th2 imbalance in AR mice.^39^, ^40^, ^41^ MiR-146a expression was found to be markedly downregulated in patients with AR. Furthermore, miR-146a was able to promote regulatory T cell (Treg) differentiation and enhance T cell activity by targeting STAT5b.^42^ Although these studies demonstrated the important role played by miRNAs in AR, their regulation by lncRNAs has not been thoroughly investigated. We found that PELATON overexpression significantly altered the expression levels of several miRNAs. Subsequent experiments demonstrated that PELATON could regulate the expression of downstream GATA3 by targeting miR-10b-5p, which underscores the pivotal role played by miR-10b-5p in AR pathogenesis. Previous studies reported that miR-10b-5p reduced the percentage of Th17 cells and the levels of key inflammatory mediators, including IgE, IL-4, IL-6, and IL-17 in AR mice.^43^ It also decreased the levels of SRSF1 in T cells from systemic lupus erythematosus (SLE) patients^44^ and inhibited IL-17A production and Th17 cell differentiation in patients with ankylosing spondylitis.^45^ Our study identified the role of miR-10b-5p in Th2 cell differentiation and activation, suggesting that it may play a crucial role in the differentiation of various Th cells under the influence of distinct upstream regulators.

LncRNAs and miRNAs regulate signaling pathways in various cell types and thereby influence the stability of immune cells and the intensity of inflammation. In AR, lncRNAs act as ceRNAs by competitively binding to miRNAs via miRNA response elements, thereby regulating target gene expression.^46^ For example, Yue et al. 2020 found that lncRNA00632, which is downregulated in the nasal mucosal tissues of patients with AR, acted as a miRNA sponge that targeted miR-498 and effectively inhibited the IL-13-triggered release of pro-inflammatory mediators.^47^ Similarly, studies have revealed that lncRNA MALAT1 competitively binds to GATA3 alongside miR-135b-5p, thereby promoting Th2 cell differentiation in patients with AR.^48^ The lncRNA JP X functions as a sponge for miR-378g, upregulating CCL5 and promoting Treg/Th17 homeostasis in AR.^49^ These findings illustrate the important role played by the ceRNA network mechanism in AR. Formation of the PELATON/miR-10b-5p/GATA3 ceRNA network regulatory mechanism presents new possibilities for finding therapeutic targets for AR.

Nevertheless, this study does have certain limitations. The generalizability of our findings is limited by the sample size, and the identified regulatory mechanism must be further verified in larger-scale studies. Transcriptome sequencing revealed that the expression levels of 51 miRNAs and 31 mRNAs were significantly altered following the overexpression of PELATON, indicating the intricate nature of how lncRNAs function. Furthermore, the number of genes potentially regulated by PELATON remains unverified, thereby necessitating further studies to elucidate the network's complexity. Although PELATON was found to regulate specific miRNAs (notably miR-10b-5p), its overall regulatory scope (particularly concerning nuclear functions and unverified target genes) remains uncharacterized. Additional research is warranted to investigate the multifaceted complexity of PELATON.

In conclusion, our study demonstrates that lncRNA PELATON promotes Th2 differentiation in patients with AR by upregulating GATA3. Mechanistically, PELATON competes with miR-10b-5p to form a ceRNA network, thereby counteracting miR-10b-5p's suppression of Th2 inflammation. These findings reveal a novel lncRNA-mediated pathway involved in AR pathogenesis and suggest PELATON as a potential biomarker and therapeutic target for AR. While inhibition of PELATON or augmentation of miR-10b-5p represents a potential therapeutic strategy for AR, achieving precisely targeted delivery while avoiding off-target effects remains challenging. Current studies indicate that nanoliposomes or exosomes are biocompatible and have low immunogenicity,^50^, ^51^ suggesting their possible use for lncRNA delivery. Our future work will focus on developing delivery methods for PELATON and miR-10b-5p, offering potential options for AR treatment.

## Abbreviations

AR, allergic rhinitis; lnc, lncRNA; GATA3, GATA binding protein 3; miR, microRNA; PBMCs, peripheral blood mononuclear cells; ceRNA, competitive endogenous RNA; UTRs, untranslated regions; SPT, skin prick test; WT, wild-type; MUT, mutant.

## Consent to participate

Informed consent was obtained from all individual participants included in the study.

## Author contribution

**Zhen Liu:** Conceptualization, Validation, Visualization, Writing - original draft. **Yumei Li:** Conceptualization, Formal analysis, Investigation. **Xiangkun Zhao**: Data curation, Validation. **Qi Sun**: Methodology, Software. **Yu Zhang:** Project administration, Writing - review & editing. **Yaqi Wang:** Data curation, Formal analysis. **Han Fang:** Data curation, Formal analysis. **Yujuan Yang**: Supervision, Project administration. **Yakui Mou:** Supervision, Project administration. **Xicheng Song:** Conceptualization, Funding acquisition, Resources, Writing - review and editing. All authors contributed to the article and approved the submitted version.

## Ethics approval

This study was approved by the Institution Ethics Committee of Yantai Yuhuangding Hospital (Approval No. No.2021-421). Our experiments were performed in accordance with the Declaration of Helsinki.

## Availability of data and materials

The datasets generated during the current study are available in the GEO repository, GSE252412 (https://www.ncbi.nlm.nih.gov/geo/query/acc.cgi?acc=GSE252412).

## Confirmation of unpublished work

The manuscript is original, has not been published before, is not currently being considered for publication elsewhere.

## Declaration of Generative AI and AI-assisted technologies in the writing process

Nothing to disclose.

## Funding

This work was supported by National Natural Science Foundation of China，China (82071021, 82301281); Major Scientific and Technological Innovation Project of Shandong Province, China (2022CXGC020506); scholar project of Yantai’s “Double Hundreds Plan, China.

## Declaration of competing interest

The authors have no relevant financial or non-financial interests to disclose.

## Figures and Tables

**figs1:**
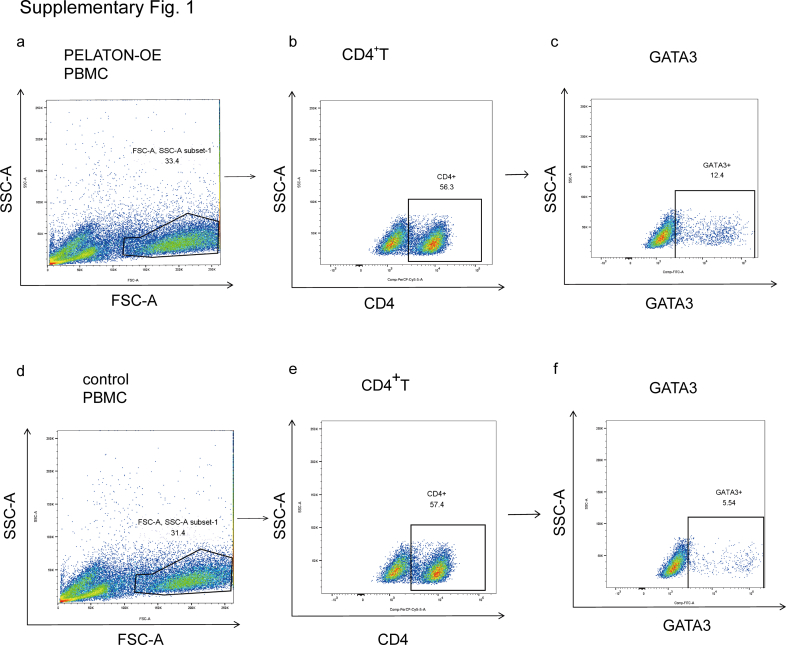


**figs2:**
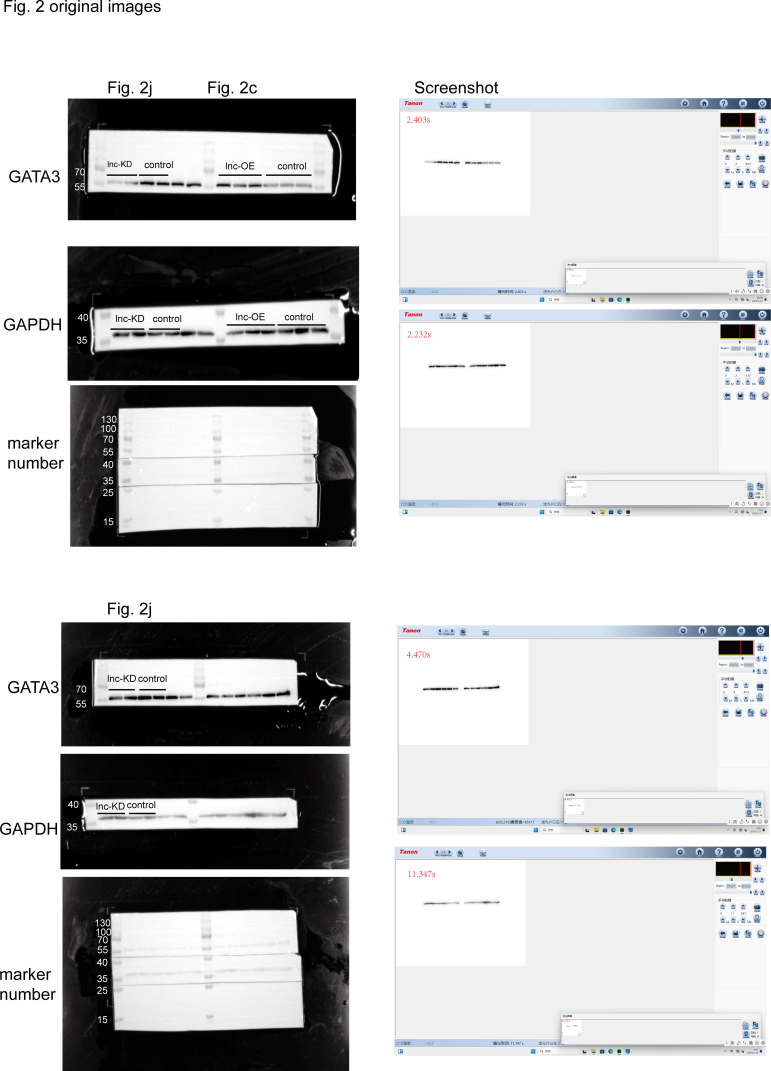


**figs3:**